# Associations between Neck Circumference, Mid-Upper Arm Circumference, Wrist Circumference, and High Blood Pressure among Lithuanian Children and Adolescents: A Cross-Sectional Study

**DOI:** 10.3390/nu16050677

**Published:** 2024-02-28

**Authors:** Ieva Stankute, Virginija Dulskiene, Renata Kuciene

**Affiliations:** Institute of Cardiology, Medical Academy, Lithuanian University of Health Sciences, Sukileliu 15, LT-50162 Kaunas, Lithuania; virginija.dulskiene@lsmu.lt (V.D.); renata.kuciene@lsmu.lt (R.K.)

**Keywords:** adolescents, children, high blood pressure, mid-upper arm circumference, neck circumference, wrist circumference

## Abstract

(1) Background: High blood pressure (HBP) and obesity are significant and growing public health issues worldwide. Our study aimed to evaluate the associations of neck circumference (NC), mid-upper arm circumference (MUAC), and wrist circumference (WrC) with HBP among Lithuanian children and adolescents aged 7–17 years. (2) Methods: In this cross-sectional study, data on BP and anthropometric measurements were analysed in 3688 children and adolescents aged 7–17 years. Multivariate logistic regression analysis was used to estimate the associations between anthropometric indices and HBP. (3) Results: Overall, the prevalence rates of elevated BP and hypertension were 13.7% and 12.9%, respectively. After adjustment for age, BMI, and WC, statistically significant elevated aORs were observed for associations between greater NC, MUAC, WrC, and HBP in boys (aORs: 2.13, 2.46, and 2.48, respectively) and in girls (aORs: 2.01, 2.36, and 2.09, respectively). Moreover, per-unit increase in NC, MUAC, and WrC was also associated with greater odds of HBP in boys (aORs: 1.20, 1.21, and 1.37, respectively) and in girls (aORs: 1.10, 1.10, and 1.21, respectively). The analysed anthropometric indices presented higher area under the curve values for predicting HBP in boys than in girls. (4) Conclusions: This study suggests that higher NC, MUAC, and WrC are associated with increased odds of HBP in Lithuanian children and adolescents.

## 1. Introduction

Elevated blood pressure and hypertension have been considered significant and growing health problems in the paediatric population [1]. In recent decades, there has been a persistent increase in the prevalence of high blood pressure (HBP), which is strongly associated with obesity in children and adolescents [2]. It has been demonstrated that elevated BP in childhood is significantly related to hypertension in adulthood [3]. Research data show that individuals who consistently experience hypertension from childhood through adolescence have a 7.6-fold higher risk of developing hypertension in adulthood [4]. Hypertension is associated with target organ damage in childhood and can increase the risk of cardiovascular disease in adulthood [3,5]. Later in life, hypertension may also trigger strokes, renal problems, kidney failure, and even death [6]. The identification of HBP in children and adolescents at an early stage could play a crucial role in preventing the emergence and progression of hypertension along with its complications in adulthood.

Body mass index (BMI), waist circumference (WC), and waist-to-height ratio (WHtR) are the most widely used anthropometric indices in defining obesity and predicting the risk of non-communicable diseases in epidemiological studies and clinical practice [7]. Growing scientific evidence analysing the associations between NC and WrC and cardiometabolic alterations in children and adolescents has also been described in a scoping review by Valencia-Sosa et al. [8]. NC is an indicator of subcutaneous fat in the upper body [9] and is also a predictor of cardiovascular risk [10]. Moreover, NC is an anthropometric tool that may be useful in screening for overweight and obesity in children [11]. Data from the ACFIES study showed that NC was positively and significantly correlated with BP, fasting plasma glucose, high-sensitivity C-reactive protein, HOMA-IR, and insulin in boys and girls [12]. A cross-sectional study by Payab et al. demonstrated that in children and adolescents aged 7–18 years, elevated NC and WrC were significantly associated with different metabolic phenotypes of obesity compared to subjects with metabolically healthy non-obesity [13]. WrC, an anthropometric measure of body frame and bone size [14,15], has also been found to be related to adipose tissue dysfunction [16]. Capizzi et al. suggested that WrC could be used in the prediction of the risk of cardiovascular disease [17]. The results of the CASPIAN-V study including children and adolescents aged 7 to 18 years revealed that NC and WrC were associated with HBP, low high-density lipoprotein cholesterol levels, overweight, obesity, abdominal obesity, and metabolic syndrome, but not with higher fasting blood glucose levels, high total cholesterol, increased triacylglycerols, or elevated low-density lipoprotein cholesterol levels [14]. A systematic review and meta-analysis revealed that high WrC was associated with a 33% higher risk of metabolic syndrome in both children and adults [15]. Epidemiological studies have suggested that MUAC, a proxy for subcutaneous fat in the upper body [18], can be an accurate, simple, and reliable screening tool for identifying underweight [19], overweight, and obesity [20,21], as well as for assessing body fat distribution in children [22]. Nowak-Szczepanska et al. showed that MUAC more than BMI may reflect long-lasting socioeconomic changes influencing the growth of children and adolescents [23]. It has been demonstrated that greater MUAC was significantly associated with an increased risk of cardiometabolic disorders, hypertension, and subclinical atherosclerosis in adults [24]. However, studies on the associations of WrC and MUAC with HBP in the paediatric population are still scarce, and we could not find any such studies conducted among children and adolescents in Lithuania or in other Baltic countries. Moreover, the prevalence of HBP in Lithuanian children aged 3–7 years (21.4%) [25] and adolescents aged 12–15 years (25.7%) [26] is high. Public health strategies should definitely focus on the prevention of HBP and obesity in childhood as the main modifiable risk factors of cardiovascular disease, which could reduce the incidence of non-communicable diseases in later life as well as their long-term consequences and mortality.

Therefore, this study aimed to evaluate the associations between NC, MUAC, WrC, and HBP among Lithuanian schoolchildren aged 7–17 years. We hypothesised that greater NC, MUAC, and WrC could be positively associated with higher odds of HBP in children and adolescents.

## 2. Materials and Methods

### 2.1. Study Population

Conducted from November 2019 to March 2020, this cross-sectional study took place in Kaunas district, the second-largest district in Lithuania. A total of 3757 participants (from the 1st to the 12th grade; aged 7–18 years) were selected using a stratified two-stage cluster sampling design. The first stage of sampling included all the schools in the Kaunas district, comprising schoolchildren aged 7–18 years. The second stage involved sampling all classes from all 29 participating schools, including primary schools, pre-gymnasiums, and gymnasiums.

Approval for the study was granted by the Kaunas Regional Biomedical Research Ethics Committee at the Lithuanian University of Health Sciences on 10 June 2019 (protocol No. BE-2-42). Verbal assent was obtained from all study participants as well as written informed consent from the parent or guardian of each participant, in accordance with the principles and guidelines of the Declaration of Helsinki. The objectives of the study were thoroughly explained to all parties involved.

### 2.2. Blood Pressure Measurements

BP measurements were conducted in the morning at the subjects’ schools by a physician without wearing a white coat. Prior to the measurements, participants were instructed to refrain from engaging in physical activity and consuming energy drinks, caffeine, coffee, or black or green tea. Using an automatic BP monitor (OMRON M6; Kyoto, Japan), three blood pressure readings were taken with 5 min intervals of rest while the subjects were seated, following a 10 min rest period.

According to the Clinical Practice Guideline for the Management of High Blood Pressure in Children and Adolescents published by the American Academy of Paediatrics [27,28], normal BP was defined as BP <90th percentile for age, sex, and height (for children < 13 years) or <120/<80 mm Hg (for adolescents ≥ 13 years old); elevated BP as BP between ≥90th percentile and <95th percentile for age, sex, and height (for children < 13 years) or 120 to 129/<80 mm Hg (for adolescents ≥ 13 years old); and hypertension as BP ≥ 95th percentile for age, sex, and height (for children < 13 years) or ≥130/80 mmHg (for adolescents ≥ 13 years old). HBP for children and adolescents was defined as having elevated BP or hypertension. Pulse pressure (PP) was calculated as SBP minus DBP. Mean arterial pressure (MAP) was calculated as follows: (SBP + (2 × DBP))/3.

### 2.3. Anthropometric Measurements

Using a portable stadiometer (Marsden, HM-250P Leicester Height Measure), the height of the subjects without shoes was measured to the nearest 0.1 cm. For body weight measurement, subjects wore light clothing and no shoes, and it was recorded to the nearest 0.1 kg using an automatic BP monitor (OMRON M6; OMRON HEALTHCARE CO., LTD, Kyoto, Japan). Overweight and obesity were determined based on BMI cutoff points specific to age and sex, as proposed by the IOTF [29].

Measuring neck circumference (NC) to the nearest 0.1 cm was performed at the level of the thyroid cartilage. Subjects were in a standing position with their heads held erect and eyes facing forward. This was carried out using a flexible measuring tape. Mid-upper arm circumference (MUAC) was measured at the midpoint between the olecranon and the acromion processes to the nearest 0.1 cm. Using a tape measure, the dominant wrist circumference (WrC) was measured to the nearest 0.1 cm while the participant was seated. The measurement was taken over Lister’s tubercle of the distal radius and over the distal ulna [16,17].

Waist circumference (WC) was measured at the mid-point between the lower margin of the last palpable rib and the top of the iliac crest, and hip circumference (HC) was measured at the maximum circumference around the buttocks. WC and HC measurements were recorded to the nearest 0.5 cm using a flexible measuring tape. Using age- and sex-specific WC percentile cut-off values suggested by the NHANES III [30], abdominal obesity was defined as WC ≥ 90th percentile. 

A body shape index (ABSI) was calculated using the following formula [31]:

$$\mathrm{ABSI}=\frac{\mathrm{WC}}{{\mathrm{BMI}}^{2/3}\times {\mathrm{Height}}^{1/2}}$$

### 2.4. Statistical Analysis

The data analysis was performed using IBM SPSS for Windows, version 27.0.

Numbers and percentages represented categorical variables and were compared using the chi-square test. The Kolmogorov–Smirnov test examined the normality of continuous variable distributions. For normally distributed continuous variables, means and standard deviations (SD) were presented, and group comparisons were conducted via the *t*-test and ANOVA.

Pearson’s correlation coefficients were calculated between anthropometric parameters (NC z-score, MUAC z-score, WrC z-score, BMI z-score, and WC z-score) and BP. Age- and sex-specific NC, MUAC, and WrC percentiles (25th, 50th, 75th, and 90th) were calculated (Supplementary Figures S1–S3). The values of the anthropometric parameters (NC, MUAC, and WrC) were considered higher if they were equal to or above the 90th percentile. Separate univariate and multivariate logistic regression analyses were performed for boys, girls, and both genders collectively to assess the relationships between NC, MUAC, WrC, and HBP. In the multivariate analysis for boys and girls separately, odds ratios (ORs) were adjusted for age, BMI, and WC. In the multivariate analysis for both sexes combined, ORs were adjusted for age, sex, BMI, and WC. Receiver operating characteristic (ROC) curve analyses were performed, and areas under the curves (AUCs) of anthropometrics parameters (NC, MUAC, WrC, BMI, and WC) for predicting HBP were calculated. The AUC was interpreted as described by Nahm [32].

Statistical significance was determined for *p*-values < 0.05.

## 3. Results

Out of the 3757 schoolchildren initially enrolled, 47 were excluded due to lacking anthropometric data (weight and height), and 42 had missing WrC measurements. Individuals aged 18 years and above were omitted from the analysis due to incomplete anthropometric data (WrC or MUAC). Consequently, the final statistical analysis encompassed 3668 subjects aged 7–17 years.

The characteristics of the study participants stratified by sex are presented in Table 1. There were 3668 subjects, of whom 1928 (52.6%) were boys and 1740 (47.4%) were girls. The mean age of the study population was 10.83 ± 2.85 years (10.85 ± 2.75 years in boys and 10.81 ± 2.78 years in girls). Boys were significantly heavier and taller, and they had significantly higher mean values of HC, NC, MUAC, WC, WrC, WHtR, WHR, and ABSI. In boys, the mean values of NC, MUAC, and WrC were, respectively, 29.68 ± 3.22 cm, 22.13 ± 3.43 cm, and 14.52 ± 1.60 cm, while in girls, they were, respectively, 28.12 ± 2.49 cm, 21.53 ± 2.97 cm, and 13.85 ± 1.34 cm. Boys exhibited significantly higher mean values of SBP and PP and lower mean values of DBP compared to girls. However, there were no notable differences in mean age, BMI, or MAP between the two groups.

The overall prevalence of elevated BP and hypertension was 13.7% (14.7% for boys and 12.6% for girls) and 12.9% (14.5% for boys and 11.1% for girls), respectively (Table 2). Boys were more likely to have HBP compared to girls (*p* < 0.05). Overall, 14.2% of the participants (12.9% of boys and 15.6% of girls) had NC ≥ 90th percentile, 10.4% (10.4% of boys and 10.5% of girls) had MUAC ≥ 90th percentile, and 10.8% (10.8% of boys and 10.8% of girls) had WrC ≥ 90th percentile. According to the IOTF criteria, the overall prevalence of overweight and obesity was 16.4% (17.8% for boys and 14.7% for girls) and 5.8% (6.9% for boys and 4.7% for girls), respectively. According to the criteria of the NHANES III, the prevalence of abdominal obesity (based on WC ≥ 90th percentile) was 5.3% (6.3% in boys and 4.1% in girls). Subjects with elevated BP and hypertension had significantly higher mean values for age, weight, height, HC, NC, MUAC, WrC, WC, BMI, WHtR, SBP, DBP, MAP, and PP compared to normotensive subjects. In boys, the mean values of age, weight, height, HC, NC, MUAC, WrC, WC, and BMI were significantly higher in the hypertensive group than in the group with elevated BP, but in girls, no significant difference between these groups in the mean age or the above-mentioned anthropometric parameters (except for WrC, WHR, and WHtR) was found.

Pearson’s correlation coefficients between the NC z-score, the MUAC z-score, the WrC z-score, the BMI z-score, the WC z-score, and BP are shown in Table 3. The z-scores of the anthropometric parameters positively and significantly correlated with BP in boys and in girls, but the correlations of the NC z-score, the MUAC z-score, the WrC z-score, the WC z-score, and the BMI z-score with SBP, MAP, and PP in boys were higher than in girls, while the correlation coefficients of the NC z-score, the MUAC z-score, the WrC z-score, and the BMI z-score with DBP were higher in girls. The NC z-score correlated significantly with the BMI z-score (for boys, r = 0.735; for girls, r = 0.750; for all participants, r = 0.722; all *p* values were <0.001) and the WC z-score (for boys, r = 0.786; for girls, r = 0.733; for all participants, r = 0.775; all *p* values were <0.001). The MUAC z-score correlated significantly with the BMI z-score (for boys, r = 0.849; for girls, r = 0.839; for all participants, r = 0.843; all *p* values were <0.001) and the WC z-score (for boys, r = 0.868; for girls, r = 0.803; for all participants, r = 0.842; all *p* values were <0.001). The WrC z-score correlated significantly with the BMI z-score (for boys, r = 0.755; for girls, r = 0.725; for all participants, r = 0.731; all *p* values were <0.001) and the WC z-score (for boys, r = 0.779; for girls, r = 0.709; for all participants, r = 0.762; all *p* values were <0.001).

Univariate analysis revealed that participants with NC, MUAC, or WrC equal to or above the 90th percentile had significantly higher odds of having HBP, if compared to the subjects with the above-mentioned anthropometric indicators below the 90th percentile (Table 4).

The multivariate logistic regression analysis, after adjustment for age, BMI, and WC, demonstrated significant associations between NC ≥ 90th, MUAC ≥ 90th, WrC ≥ 90th, and increased odds of HBP in boys (aORs: 2.13, 2.46, 2.48, respectively (*p* < 0.001)) and in girls (aOR 2.01, 2.36, 2.09, respectively (*p* < 0.001)). Moreover, per-unit increases in NC, MUAC, and WrC were associated with greater odds of HBP in boys (aORs: 1.20, 1.21, 1.37, respectively (*p* < 0.001)) and in girls (aORs: 1.10, 1.10, 1.21, respectively (*p* < 0.001)). In general, the magnitudes of the aORs were slightly greater in boys than in girls. The results of the adjusted models also remained statistically significant in both sexes combined.

Table 5 shows the results of the ROC analysis for anthropometric parameters (NC, MUAC, WrC, BMI, and WC) for the prediction of HBP in boys and in girls separately. The highest AUCs were found for MUAC in boys aged 7–12 years (AUC = 0.757) and for NC in boys aged 13–17 years (AUC = 0.762). The AUC values of anthropometric parameters were greater in boys than in girls. Also, the AUC values were found to be higher in 7–12-year-old girls than in 13–17-year-old girls (Supplementary Figure S4).

## 4. Discussion

In the current study, 26.6% of subjects had HBP (13.7%—elevated BP, and 12.9%—hypertension), and the prevalence of HBP was higher among boys than among girls. Such findings were also demonstrated in other studies [33,34]. In this study, we presented the associations of the anthropometric parameters—NC, MUAC, and WrC—with HBP in Lithuanian schoolchildren.

Our results, similar to those obtained in other studies, showed that NC significantly correlated with SBP, DBP [12,35,36,37], MAP [38], BMI, and WC [36,39,40]. However, data from studies of paediatric populations in Mexico [40] and Greece [41] showed that NC positively and significantly correlated with SBP in boys and in girls, but with DBP, only in girls. In the Brazilian Metabolic Syndrome Study, NC positively and significantly correlated with SBP and DBP in adolescents of both sexes, except in prepubertal girls [39]. Our study also confirmed that larger NC was associated with increased odds of HBP in children and adolescents, and this is in agreement with the findings obtained by other researchers [42]. The results from a systematic review and meta-analysis of studies among children (aged < 18 years) in different countries showed that NC significantly correlated with SBP and DBP; additionally, higher NC was associated with the risk of hypertension (OR = 1.35; 95% CI: 1.05–1.75) [43]. In our prior study, we found high NC alone, but especially in combination with obesity and abdominal obesity, was related to increased odds of HBP among 12–15-year-old adolescents [44]. Epidemiological studies of children and adolescents have shown that NC is related to some cardiometabolic risk factors [14,36,41]. Castro-Piñero et al. showed that NC was positively associated with selected cardiovascular risk factors (BP, triglycerides, and HOMA) and inflammatory markers in Spanish children and adolescents [36]. A meta-analysis by Ataie-Jafari et al. revealed a positive association between high NC and some cardiometabolic risk factors (fasting blood sugar, total cholesterol, and triglyceride concentration) in children [10]. Peña-Vélez et al. reported that NC statistically significantly correlated with SBP, WC, WHtR, alanine aminotransferase levels, HOMA-IR, and insulin and was also associated with non-alcoholic fatty liver disease in children with obesity [45].

It was shown that larger NC can identify obesity and predict cardiometabolic risk in children and adolescents [7]. However, few studies examined the accuracy of NC in predicting HBP in paediatric populations. In the present study, the ROC analysis showed that the NC had greater AUC values for boys (ranging from 0.728 to 0.762) than for girls (ranging from 0.647 to 0.665) in predicting HBP. A study by Nafiu et al. [42] revealed that the AUC values of NC were also higher in boys (AUC 0.75) than in girls (AUC 0.72) in identifying children and adolescents with HBP. However, in our study, NC demonstrated lower predictive accuracy among girls.

NC measurement is a simple, low-cost, time-saving, and convenient measure. It is not affected by diet and respiratory conditions [46] and has very good inter- and intra-rater reliability [47]. However, different NC measurements at different anatomical sites are performed in different studies, for instance, at the level of the thyroid cartilage [40,42] or below the thyroid cartilage [12,36,41]. Despite differences in measuring approaches, NC proves to be a useful marker of fat accumulation in the neck area resulting from significant and prolonged weight gain, aiding in the detection of HBP [8]. Additional large-scale studies are needed to confirm specific NC cut-off values for paediatric clinical practice.

Our study showed that WrC correlated significantly with SBP and DBP in boys and girls, and these findings are in line with existing data from other studies [14,48]. The results of our study also showed significant associations between higher WrC and increased odds of HBP in children and adolescents. These results are consistent with those obtained in previous studies where researchers also found significant relationships [14,48]. Kelishadi et al. found that WrC was significantly associated with HBP (aOR = 1.26) in children and adolescents aged 7–18 years [14]. In a cross-sectional study by Kajale et al., Indian children and adolescents aged 6–18 years with large WrCs had a 1.26-fold greater risk of hypertension [48]. Ejtahed et al. showed that high WrC was associated with an increased risk score of metabolic syndrome (aOR = 1.5) in the paediatric population [38].

Capizzi et al. found a correlation between WrC, transversal wrist internal bone tissue area, and insulin resistance in children and adolescents with overweight/obesity [17]. Studies of children and adolescents with overweight/obesity by Zampetti et al. demonstrated that WrC, a clinical marker of insulin resistance, was associated with elevated SBP [49] and was also significantly related to left ventricular hypertrophy [50]. WrC was also significantly associated with the adiponectin–leptin ratio and with the metabolic syndrome score in youths with obesity [16]. WrC, BP, and total cholesterol in adolescents have been found to be predictors of hypertension in adulthood [51]. WrC can be used to determine body frame size, which may reflect cardiometabolic risk and obesity [52].

WrC serves as a straightforward, non-intrusive anthropometric measurement with easy measurability [50], demonstrating excellent intra- and inter-operator reliability and reproducibility [53]. Nevertheless, across diverse studies, different WrC measurements are performed at different anatomical sites: distal to the prominences of the radial and ulnar bones [14,51], over the Lister tubercle of the distal radius and over the distal ulna [16,17], and at the most prominent aspect of the radial styloid process [48]. Moreover, there is no internationally accepted sex- and age-specific WrC cut-off value to identify subjects with an increased cardiometabolic risk. Additionally, there is limited evidence about the performance of WrC in predicting HBP, which needs further research. In a study by Khadilkar et al., the cut-off of WrC for identifying the risk of hypertension in Indian children and adolescents aged 3–18 years was the 70th percentile [54]. Ahilan et al. observed that the 97th percentile of WrC predicted an increased cardiometabolic risk in 5–17-year-old Indian children with overweight/obesity [55]. In the present study, the ROC analysis showed that WrC had a moderate ability in boys and a low ability in girls to predict HBP.

In the current study, we found significant correlations between MUAC and both SBP and DBP, which is consistent with the findings of Mazicioglu et al. [56], Ma et al. [57], and Mphahlele et al. [58]. However, in the Sardinian Hypertensive Adolescent Research Programme study, MUAC significantly correlated with SBP but not with DBP [59]. Our study demonstrated that greater MUAC was associated with higher odds of HBP. Results from a cross-sectional study by Hou and colleagues have also shown a relationship between larger MUAC and hypertension in men and women [24]. However, there is a scarcity of studies investigating the association between larger MUAC and HBP in the paediatric population. In the present study, the ROC analysis showed that AUC values of MUAC ranged from 0.754 to 0.757 for boys and from 0.642 to 0.678 for girls. These findings are in line with the data of a cross-sectional study conducted in Turkey [56], where the AUC values were 0.747 for boys aged 11–14 years and <0.700 for girls aged 11–14 years and 14–17 years. 

MUAC stands out as a cost-effective, quick, non-invasive, and easily measurable anthropometric index [60], and it can be employed in research studies to identify individuals with central obesity and insulin resistance in type 2 diabetes [61]. In comparison to the BMI method, MUAC presents clear benefits, particularly in its straightforward measurement without requiring mathematical derivation [62]. In an observational multinational cross-sectional study including 9–11-year-old children from 12 countries, MUAC was demonstrated as an alternative screening measure for obesity [63]. Matjuda et al. analysed the relationships of obesity and hypertension with the risk of renal–cardiovascular disease in children aged 6–9 years and found that asymmetric dimethylarginine positively correlated with MUAC, while creatinine was positively associated with other obesity measures, namely NC and WC [64].

The available evidence indicates a connection between elevated adiposity in the upper body and heightened liberation of free fatty acids, thereby playing a role in the adverse health outcomes associated with obesity [65]. Research data have demonstrated that increased volumes of upper body subcutaneous fat and larger NCs are associated with elevated risks of cardiovascular factors, suggesting a potentially higher metabolic risk compared to abdominal visceral fat [66,67]. In a cross-sectional study by Arias-Tellez et al., larger total neck adipose tissue volume and NC were associated with a higher cardiometabolic risk and a proinflammatory status in young adults. It was also demonstrated that NC was correlated with BMI, fat mass, and visceral adipose tissue [68]. Both abdominal visceral adipose tissue and subcutaneous adipose tissue (measured by dual-energy X-ray absorptiometry) were significantly related to adverse cardiometabolic risk factors in a paediatric population [69]. Chomtho et al. identified that MUAC is a good predictor of the total body fat mass but not of the fat-free mass in children [70]. A cross-sectional study of Indian children and adolescents found that WrC and NC positively and significantly correlated with body fat percentage [71]. A cross-sectional study of Chinese children and adolescents demonstrated that the odds of having elevated BP levels increased with increasing levels of body fat percentage [72]. In a cross-sectional study by He et al., children and adolescents with high body fat percentage had an increased risk of cardiometabolic risk factors (including dyslipidaemia, hyperglycaemia, and HBP) [33]. The mechanisms underlying obesity-related hypertension are complex and include stimulation of the renin–angiotensin–aldosterone system, sympathetic nervous system overactivation, leptin resistance and hyperleptinemia, insulin resistance, and functional and structural renal changes [73].

The strength of our study was a wide age range (7–17 years) of Lithuanian schoolchildren. This is the first cross-sectional study to assess the associations of higher MUAC and WrC with HBP in Lithuanian children and adolescents. Nevertheless, it is essential to acknowledge certain limitations of our research. The geographical focus of the study on a singular district may limit the generalisability of our findings. Moreover, the cross-sectional design used in our study inherently lacks the capacity to establish causal relationships, posing a difficulty in interpreting the identified associations. During this study, skinfold measurements were not conducted, and data regarding the pubertal status of the subjects were not collected. Additionally, there was no examination of blood or biochemical parameters. The validation of the diagnosis of elevated BP was primarily reliant on a single visit, assessing the average of three BP readings. Moreover, differences in the age of the examined schoolchildren, sample sizes, BP, and methodologies of the anthropometric measurements in children have also made it difficult to make comparisons across studies. Despite the above-mentioned limitations, the present study provided additional insights into the associations between higher values of these anthropometric parameters and increased odds of HBP in children and adolescents.

## 5. Conclusions

Our results showed a high prevalence of HBP among 7–17-year-old Lithuanian schoolchildren. Larger NCs, MUACs, and WrCs were associated with greater odds of HBP in children and adolescents. Moreover, the accuracy of the analysed anthropometric parameters in predicting HBP was higher in boys than in girls. More large-scale multi-ethnic paediatric population studies are needed to obtain more comprehensive results.

## Figures and Tables

**Table 1:** Characteristics of the study subjects by sex.

Variables	Total (n = 3668)	Boys (n = 1928)	Girls (n = 1740)	*p* *
Age (years)	10.83 ± 2.76	10.85 ± 2.75	10.81 ± 2.78	0.528
Weight (kg)	42.98 ± 16.08	44.27 ± 17.33	41.56 ± 14.44	<0.001
Height (cm)	149.27 ± 16.98	150.68 ± 18.07	147.71 ± 15.53	<0.001
HC (cm)	78.72 ± 11.40	79.20 ± 11.56	78.19 ± 11.21	0.017
NC (cm)	28.94 ± 3.00	29.68 ± 3.22	28.12 ± 2.49	<0.001
MUAC (cm)	21.84 ± 3.23	22.13 ± 3.43	21.53 ± 2.97	<0.001
WC (cm)	62.88 ± 9.95	64.60 ± 10.73	60.98 ± 8.61	<0.001
WrC (cm)	14.21 ± 1.52	14.52 ± 1.60	13.85 ± 1.34	<0.001
ABSI	0.074 ± 0.006	0.075 ± 0.006	0.072 ± 0.007	<0.001
BMI (kg/m^2^)	18.62 ± 3.71	18.76 ± 3.82	18.46 ± 3.59	0.055
WHR	0.801 ± 0.07	0.816 ± 0.06	0.784 ± 0.07	<0.001
WHtR	0.422 ± 0.05	0.43 ± 0.05	0.414 ± 0.05	<0.001
SBP (mm Hg)	109.06 ± 13.40	110.20 ± 14.07	107.80 ± 12.51	<0.001
DBP (mm Hg)	62.35 ± 8.16	61.81 ± 8.09	62.94 ± 8.21	<0.001
MAP (mm Hg)	77.92 ± 8.58	77.94 ± 8.63	77.90 ± 8.52	0.873
PP (mm Hg)	46.71 ± 11.76	48.39 ± 12.56	44.86 ± 10.50	<0.001

* Boys versus girls. ABSI—a body shape index, BMI—body mass index, DBP—diastolic blood pressure, HC—hip circumference, MAP—mean arterial pressure, MUAC—mid-upper arm circumference, NC—neck circumference, PP—pulse pressure, SBP—systolic blood pressure, WC—waist circumference, WHR—waist–hip ratio, WrC—wrist circumference, WHtR—waist-to-height ratio. Data are presented as the mean ± SD.

**Table 2:** Characteristics of the study participants according to BP level.

Variables	Normal BP	Elevated BP	Hypertension	*p* *
***Boys***				
NC percentile categories:				
<90th	1257 (92.0)	220 (77.7) ^§^	202 (72.4) ^§^	<0.001
≥90th	109 (8.0)	63 (22.3) ^§^	77 (27.6) ^§^	
MUAC percentile categories:				<0.001
<90th	1279 (93.6)	239 (84.5) ^§^	210 (75.3) ^§,#^	
≥90th	87 (6.4)	44 (15.5) ^§^	69 (24.7) ^§,#^	
WrC percentile categories:				<0.001
<90th	1274 (93.3)	241 (85.2) ^§^	205 (73.5) ^§,#^	
≥90th	92 (6.7)	42 (14.8) ^§^	74 (26.5) ^§,#^	
WC percentile categories:				<0.001
<90th	1310 (95.9)	261 (92.2) ^§^	235 (84.2) ^§,#^	
≥90th	56 (4.1)	22 (7.8) ^§^	44 (15.8) ^§,#^	
BMI categories:				<0.001
Normal weight	1107 (81.0)	187 (66.1) ^§^	157 (56.3) ^§,#^	
Overweight	203 (14.9)	71 (25.1) ^§^	70 (25.1) ^§^	
Obesity	56 (4.1)	25 (8.8) ^§^	52 (18.6) ^§,#^	
BMI categories:				<0.001
Normal weight	1107 (81.0)	187 (66.1) ^§^	157 (56.3) ^§,#^	
Overweight/obesity	259 (19.0)	96 (33.9) ^§^	122 (43.7) ^§,#^	
Weight (kg)	38.44 ± 13.11	55.16 ± 16.27 ^a^	61.78 ± 19.37 ^a,b^	<0.001
Height (cm)	145.30 ± 15.30	162.10 ± 16.53 ^a^	165.42 ± 17.69 ^a,b^	<0.001
HC (cm)	75.24 ± 9.49	87.37 ± 9.39 ^a^	90.29 ± 11.21 ^a,b^	<0.001
NC (cm)	28.58 ± 2.53	31.87 ± 2.99 ^a^	32.84 ± 3.27 ^a,b^	<0.001
MUAC (cm)	20.99 ± 2.76	24.40 ± 2.97 ^a^	25.40 ± 3.57 ^a,b^	<0.001
WrC (cm)	14.01 ± 1.37	15.58 ± 1.35 ^a^	15.97 ± 1.47 ^a,b^	<0.001
WC (cm)	61.33 ± 8.63	70.88 ± 9.91 ^a^	74.26 ± 12.11 ^a,b^	<0.001
ABSI	0.0753 ± 0.006	0.0747 ± 0.007 ^a^	0.0739 ± 0.007 ^a^	<0.001
BMI (kg/m^2^)	17.70 ± 3.10	20.56 ± 3.56 ^a^	22.10 ± 4.60 ^a,b^	<0.001
WHR	0.82 ± 0.06	0.81 ± 0.06 ^a^	0.82 ± 0.06	0.069
WHtR	0.42 ± 0.05	0.44 ± 0.06 ^a^	0.45 ± 0.07 ^a^	<0.001
SBP (mm Hg)	103.37 ± 8.90	121.24 ± 5.54 ^a^	132.41 ± 10.21 ^a,b^	<0.001
DBP (mm Hg)	59.59 ± 6.89	64.88 ± 7.16 ^a^	69.57 ± 8.61 ^a,b^	<0.001
MAP (mm Hg)	77.63 ± 6.31	88.10 ± 4.24 ^a^	95.46 ± 6.35 ^a,b^	<0.001
PP (mm Hg)	43.78 ± 9.25	56.36 ± 10.13 ^a^	62.84 ± 13.80 ^a,b^	<0.001
***Girls***				
NC percentile categories:				<0.001
<90th	1172 (88.3)	170 (77.3) ^§^	127 (65.8) ^§,#^	
≥90th	155 (11.7)	50 (22.7) ^§^	66 (34.2) ^§,#^	
MUAC percentile categories:				<0.001
<90th	1237 (93.2)	184 (83.6) ^§^	137 (71.0) ^§,#^	
≥90th	90 (6.8)	36 (16.4) ^§^	56 (29.0) ^§,#^	
WrC percentile categories:				<0.001
<90th	1229 (92.6)	184 (83.6) ^§^	139 (72.0) ^§,#^	
≥90th	98 (7.4)	36 (16.4) ^§^	54 (28.0) ^§,#^	
WC percentile categories:				<0.001
<90th	1291 (97.3)	211 (95.9)	166 (86.0) ^§,#^	
≥90th	36 (2.7)	9 (4.1)	27 (14.0) ^§,#^	
BMI categories:				<0.001
Normal weight	1125 (84.8)	161 (73.2) ^§^	117 (60.6) ^§,#^	
Overweight	162 (12.2)	44 (20.0) ^§^	50 (25.9) ^§^	
Obesity	40 (3.0)	15 (6.8) ^§^	26 (13.5) ^§,#^	
BMI categories:				<0.001
Normal weight	1125 (84.8)	161 (73.2) ^§^	117 (60.6) ^§,#^	
Overweight/obesity	202 (15.2)	59 (26.8) ^§^	76 (39.4) ^§,#^	
Weight (kg)	38.94 ± 13.05	49.64 ± 15.91 ^a^	50.34 ± 14.99 ^a^	<0.001
Height (cm)	145.70 ± 15.27	154.75 ± 14.71 ^a^	153.46 ± 14.48 ^a^	<0.001
HC (cm)	76.10 ± 10.33	84.94 ± 12.03 ^a^	84.85 ± 10.46 ^a^	<0.001
NC (cm)	27.70 ± 2.33	29.27 ± 2.51 ^a^	29.70 ± 2.54 ^a^	<0.001
MUAC (cm)	21.00 ± 2.71	22.99 ± 3.12 ^a^	23.52 ± 3.14 ^a^	<0.001
WrC (cm)	13.63 ± 1.24	14.43 ± 1.36 ^a^	14.74 ± 1.38 ^a, b^	<0.001
WC (cm)	59.46 ± 7.58	65.05 ± 9.38 ^a^	66.78 ± 10.30 ^a^	<0.001
ABSI	0.0729 ± 0.007	0.0712 ± 0.007 ^a^	0.0714 ± 0.007 ^a^	<0.001
BMI (kg/m^2^)	17.81 ± 3.13	20.18 ± 4.01 ^a^	21.00 ± 4.23 ^a^	<0.001
WHR	0.79 ± 0.07	0.77 ± 0.07 ^a^	0.79 ± 0.08 ^b^	0.001
WHtR	0.41 ± 0.05	0.42 ± 0.05 ^a^	0.44 ± 0.07 ^a, b^	<0.001
SBP (mm Hg)	103.20 ± 9.03	118.78 ± 7.76 ^a^	126.89 ± 11.57 ^a,b^	<0.001
DBP (mm Hg)	60.61 ± 6.94	68.26 ± 6.05 ^a^	72.90 ± 8.18 ^a,b^	<0.001
MAP (mm Hg)	78.16 ± 6.64	89.08 ± 4.32 ^a^	95.14 ± 6.74 ^a,b^	<0.001
PP (mm Hg)	42.59 ± 8.58	50.53 ± 10.70 ^a^	53.99 ± 14.24 ^a,b^	<0.001
***Boys***				
Age (years):				<0.001
7–12	1159 (84.8)	119 (42.0) ^§^	116 (41.6) ^§^	
13–17	207 (15.2)	164 (58.0) ^§^	163 (58.4) ^§^	
Age (years)	10.07 ± 2.33	12.48 ± 2.60 ^a^	13.05 ± 2.88 ^a,b^	<0.001
***Girls***				
Age (years):				<0.001
7–12	1034 (77.9)	112 (50.9) ^§^	117 (60.6) ^§,#^	<0.001
13–17	293 (22.1)	108 (49.1) ^§^	76 (39.4) ^§,#^	<0.001
Age (years)	10.46 ± 2.68	12.10 ± 2.90 ^a^	11.68 ± 2.69 ^a^	<0.001

ABSI—a body shape index, BMI—body mass index, DBP—diastolic blood pressure, HC—hip circumference, MAP—mean arterial pressure, MUAC—mid-upper arm circumference, NC—neck circumference, PP—pulse pressure, SBP—systolic blood pressure, WC—waist circumference, WHR—waist–hip ratio, WrC—wrist circumference, WHtR—waist-to-height ratio. ^§^ *p* < 0.05 vs. NBP group (z test). ^#^ *p* < 0.05 vs. elevated BP group (z test). ^a^ *p* < 0.05 vs. NBP group. ^b^ *p* < 0.05 vs. elevated BP group. * Significant difference between three groups.

**Table 3:** Pearson’s correlation coefficients between z-scores of anthropometric parameters and BP.

		NC z-Score	MUAC z-Score	WrC z-Score	BMI z-Score	WC z-Score
SBP (mm Hg)	Boys	0.701 **	0.671 **	0.679 **	0.554 **	0.620 **
	Girls	0.575 **	0.562 **	0.558 **	0.503 **	0.506 **
	Total	0.649 **	0.630 **	0.632 **	0.533 **	0.579 **
DBP (mm Hg)	Boys	0.329 **	0.346 **	0.317 **	0.330 **	0.350 **
	Girls	0.363 **	0.366 **	0.344 **	0.381 **	0.332 **
	Total	0.309 **	0.345 **	0.303 **	0.349 **	0.321 **
MAP (mm Hg)	Boys	0.586 **	0.581 **	0.567 **	0.507 **	0.555 **
	Girls	0.514 **	0.511 **	0.494 **	0.491 **	0.461 **
	Total	0.535 **	0.547 **	0.521 **	0.499 **	0.505 **
PP (mm Hg)	Boys	0.574 **	0.529 **	0.557 **	0.408 **	0.470 **
	Girls	0.401 **	0.383 **	0.395 **	0.301 **	0.343 **
	Total	0.525 **	0.478 **	0.510 **	0.365 **	0.437 **

** Correlation is significant at the level of 0.01 (2-tailed). BMI—body mass index, DBP—diastolic blood pressure, MAP—mean arterial pressure, MUAC—mid-upper arm circumference, NC—neck circumference, PP—pulse pressure, SBP—systolic blood pressure, WC—waist circumference, WrC—wrist circumference.

**Table 4:** Associations between NC, MUAC, WrC, and HBP (univariate and multivariate analyses).

Variables	Boys		Girls		Total	
	OR (95% CI)	aOR^1^ (95% CI)	OR (95% CI)	aOR^1^ (95% CI)	OR (95% CI)	aOR^2^ (95% CI)
NC percentile categories:						
<90th	1.00	1.00	1.00	1.00	1.00	1.00
≥90th	3.83 (2.91–5.03)	2.13 (1.47–3.07)	2.95 (2.25–3.88)	2.01 (1.43–2.82)	3.28 (2.71–3.97)	2.12 (1.66–2.72)
NC (a continuous variable)						
Per-unit increase	1.25 (1.23–1.28)	1.20 (1.16–1.24)	1.16 (1.14–1.19)	1.10 (1.07–1.14)	1.21 (1.19–1.22)	1.16 (1.14–1.19)
MUAC percentile categories:						
<90th	1.00	1.00	1.00	1.00	1.00	1.00
≥90th	3.70 (2.74–4.99)	2.46 (1.59–3.80)	3.94 (2.87–5.40)	2.36 (1.56–3.58)	3.78 (3.05–4.70)	2.33 (1.73–3.14)
MUAC (a continuous variable)						
Per-unit increase	1.22 (1.20–1.25)	1.21 (1.17–1.24)	1.14 (1.12–1.16)	1.10 (1.07–1.13)	1.19 (1.17–1.20)	1.16 (1.13–1.18)
WrC percentile categories:						
<90th	1.00	1.00	1.00	1.00	1.00	1.00
≥90th	3.60 (2.68–4.83)	2.48 (1.67–3.68)	3.49 (2.56–4.77)	2.09 (1.41–3.11)	3.53 (2.85–4.37)	2.23 (1.69–2.95)
WrC (a continuous variable)						
Per-unit increase	1.53 (1.47–1.60)	1.37 (1.29–1.45)	1.33 (1.27–1.39)	1.21 (1.14–1.28)	1.43 (1.38–1.47)	1.31 (1.26–1.36)

BMI—body mass index, MUAC—mid-upper arm circumference, NC—neck circumference, WC—waist circumference, WrC—wrist circumference. OR—odds ratio; aOR^1^—adjusted odds ratio for age, BMI, and WC; aOR^2^—adjusted odds ratio for age, sex, BMI, and WC; CI—confidence interval. All results were significant at *p* < 0.001.

**Table 5:** Area under ROC curves (95% CI) of anthropometric indices to predict HBP.

Sex/Age (Years)	Indices	AUC	(95% CI)	SE	*p* Value
***Boys***					
7–12	NC	0.728	0.692–0.764	0.018	<0.001
	MUAC	0.757	0.722–0.792	0.018	<0.001
	WrC	0.732	0.697–0.767	0.018	<0.001
	BMI	0.725	0.690–0.760	0.018	<0.001
	WC	0.744	0.708–0.780	0.018	<0.001
13–17	NC	0.762	0.721–0.802	0.021	<0.001
	MUAC	0.754	0.713–0.796	0.021	<0.001
	WrC	0.745	0.702–0.787	0.022	<0.001
	BMI	0.697	0.652–0.743	0.023	<0.001
	WC	0.706	0.660–0.751	0.022	<0.001
***Girls***					
7–12	NC	0.665	0.626–0.705	0.020	<0.001
	MUAC	0.678	0.639–0.716	0.020	<0.001
	WrC	0.676	0.637–0.715	0.020	<0.001
	BMI	0.674	0.636–0.712	0.020	<0.001
	WC	0.679	0.639–0.718	0.020	<0.001
13–17	NC	0.647	0.596–0.698	0.026	<0.001
	MUAC	0.642	0.591–0.694	0.026	<0.001
	WrC	0.612	0.559–0.665	0.027	<0.001
	BMI	0.657	0.606–0.708	0.026	<0.001
	WC	0.634	0.582–0.685	0.026	<0.001

Data are shown as AUC (95% confidence interval). AUC—area under the receiver operating characteristic curve, BMI—body mass index, MUAC—mid-upper arm circumference, NC—neck circumference, WC—waist circumference, WrC—wrist circumference.

## Data Availability

In accordance with the Statute of the Lithuanian University of Health Sciences, the authors are unable to disclose the data supporting this study. Researchers interested in accessing the data should initiate contact with the database owner, the Lithuanian University of Health Sciences. Ethical considerations prevent the data from being publicly accessible.

## Supplementary Materials

The following supporting information can be downloaded at https://www.mdpi.com/article/10.3390/nu16050677/s1, Figure S1: Age- and sex-specific percentile values of the NC in study participants aged 7–17 years. Figure S2: Age- and sex-specific percentile values of the MUAC in study participants aged 7–17 years. Figure S3: Age- and sex-specific percentile values of the WrC in study participants aged 7–17 years. Figure S4: Area under ROC curves of anthropometric indices to predict HBP.
