# Supplementary material for: Associations between Neck Circumference, Mid-Upper Arm Circumference, Wrist Circumference, and High Blood Pressure among Lithuanian Children and Adolescents: A Cross-Sectional Study
Source: Nutrients. 2024 Feb 28;16(5):677. doi: 10.3390/nu16050677 (PMC10935390; doi:10.3390/nu16050677)
Supplement: Supplementary file 1 [file nutrients-16-00677-s001.zip › nutrients-2864063-supplementary.pdf]

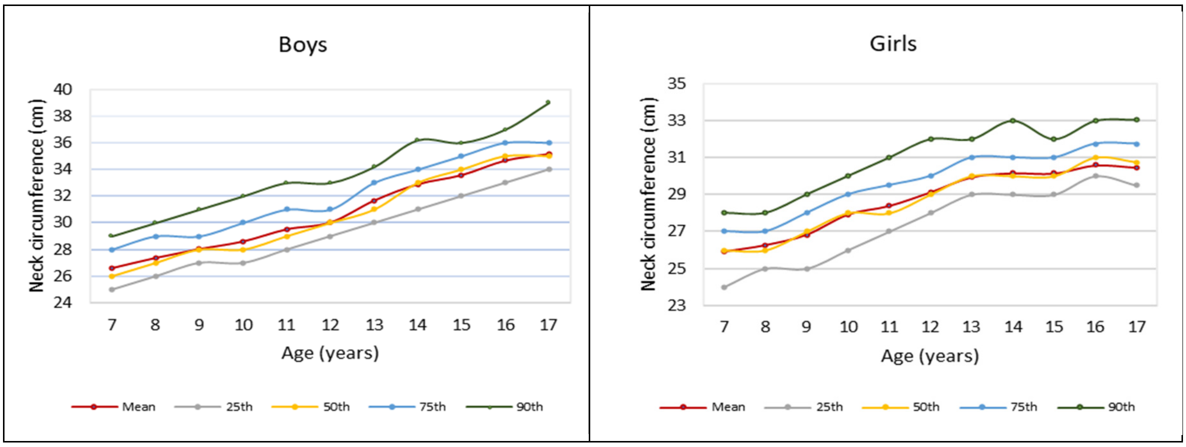

Figure S1. Age- and sex-specific percentile values of the NC in study participants aged 7–17 years.

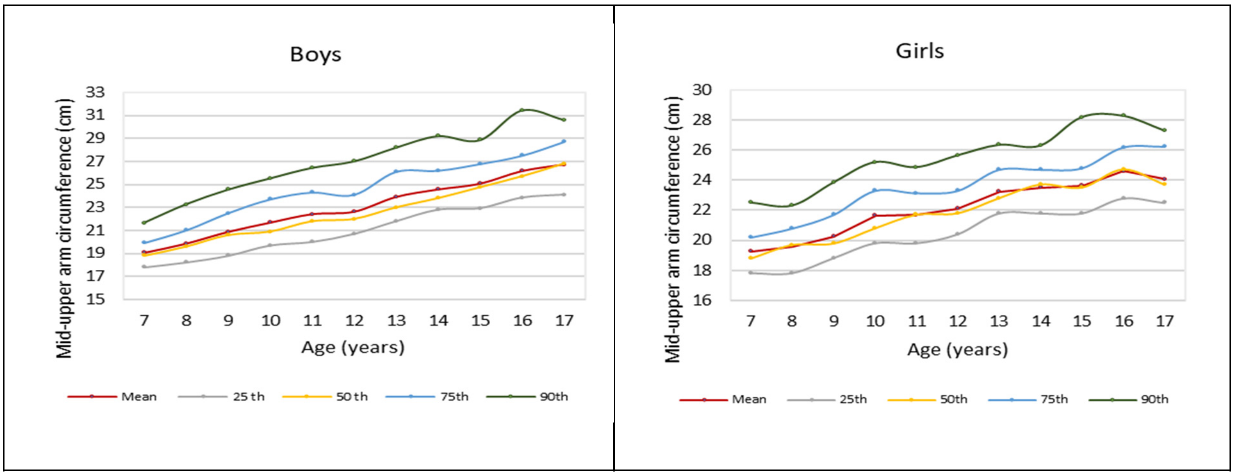

Figure S2. Age- and sex-specific percentile values of the MUAC in study participants aged 7–17 years.

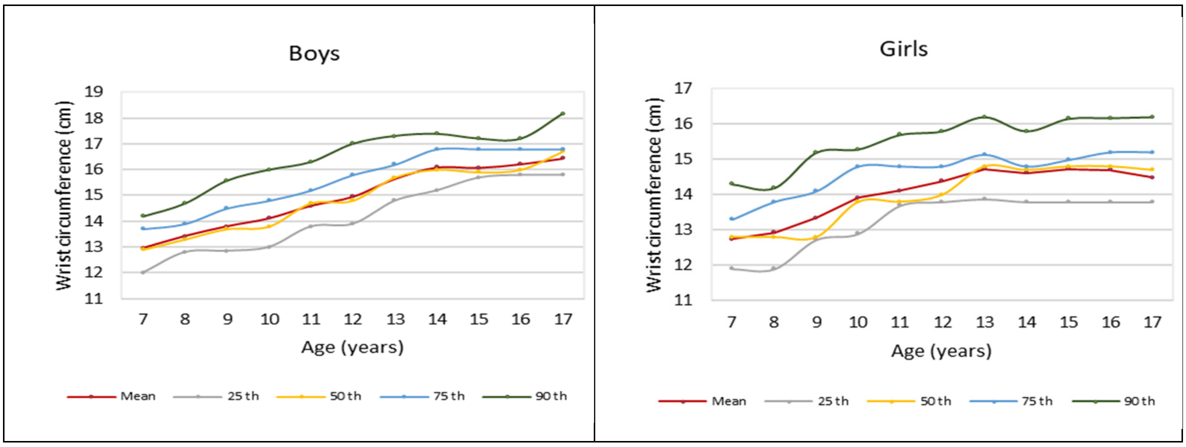

Figure S3. Age- and sex-specific percentile values of the WrC in study participants aged 7–17 years.

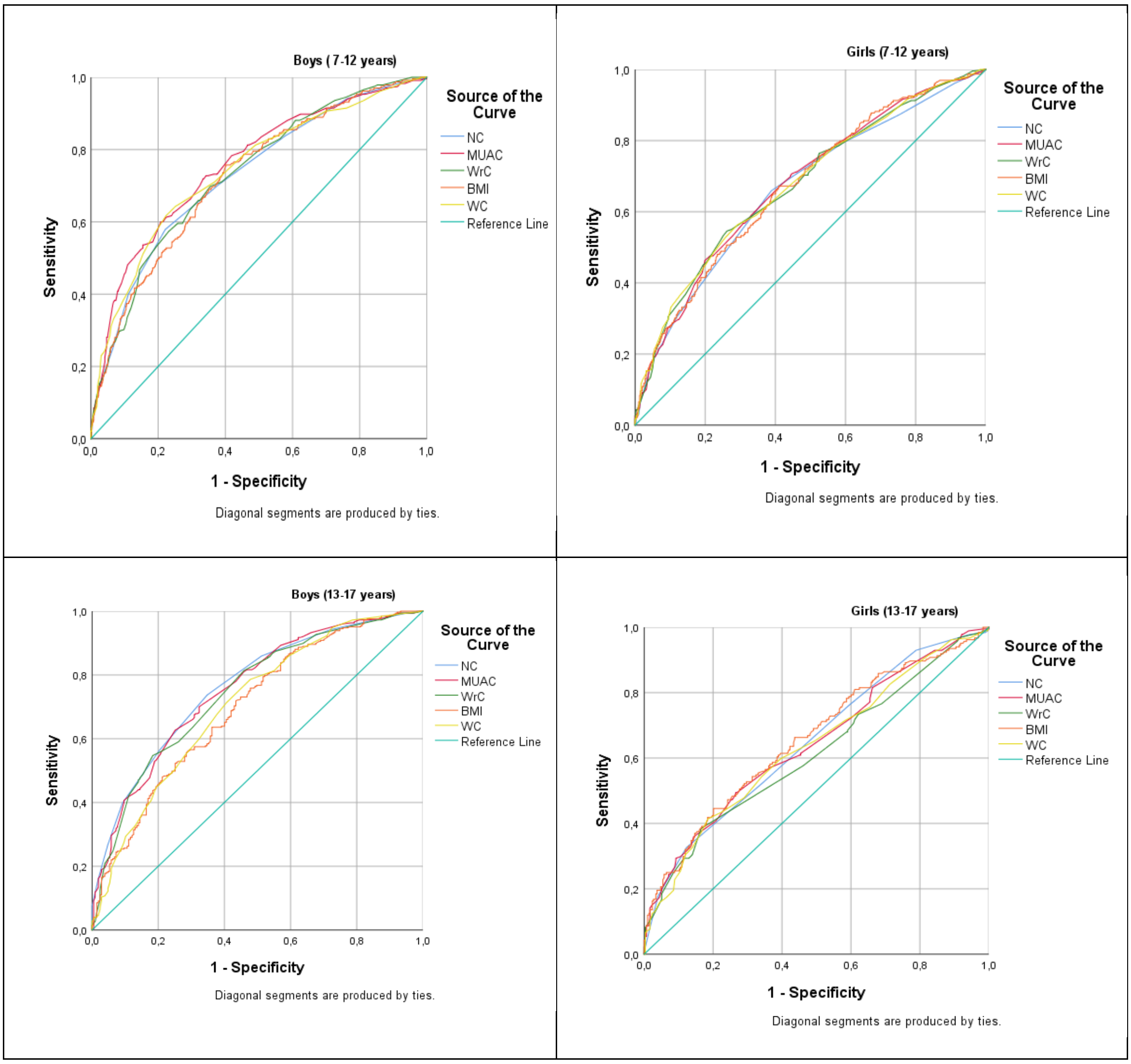

Figure S4. Area under ROC curves of anthropometric indices to predict HBP.
